# Criminal liability of medical professionals in the São Paulo State Court of Appeals

**DOI:** 10.1590/S1679-45082018AO4060

**Published:** 2018-04-06

**Authors:** Isabel de Fátima Alvim Braga, Laila Zelkcovicz Ertler, Rodrigo Moreira de Aquino, Bruno de Avilla da Fonseca e Silva, Renata Bastos Mello Pereira

**Affiliations:** 1 Fundação Oswaldo Cruz, Rio de Janeiro, RJ, Brazil; 2 Universidade Federal do Rio de Janeiro, Macaé, RJ, Brazil; 3 Hospital Municipal Miguel Couto, Rio de Janeiro, RJ, Brazil; 4 Fundação Getúlio Vargas, Rio de Janeiro, RJ, Brazil

**Keywords:** Damage liability, Medical errors/legislation & jurisprudence, Legislation, medical, Responsabilidade civil, Erros médicos/legislação & jurisprudência, Legislação médica

## Abstract

**Objective:**

To collect criminal justice data involving medical professionals in the São Paulo State Court of Appeals and to establish the number of criminal proceedings involving said professionals, the content of the accusations and the conviction rate per specialty.

**Methods:**

A keyword search was carried out in the State Appellate Court case law website with the term “medical error” for decisions rendered from January 1st, 2011 to December 31st, 2016, and the subject “criminal law” was selected.

**Results:**

A total of 34 cases met the inclusion criteria and were analyzed. Lower court's convictions accounted to 73.5% of the cases, with a slight tendency towards increase over the years. The number of cases per medical specialty was ten cases of clinical emergency, eight of obstetrics, seven of surgery, three of pediatrics (one case was related to pediatrics and obstetrics), two of orthopedic surgery, two of clinical director, one anesthesiologist and one nonemergency internal medicine physician. Among these cases, 6 were related to bodily injury, 26 to homicide and 2 criminal contempt.

**Conclusion:**

The physicians most exposed to medical error were from surgical specialties, probably due to the higher rate of complications associated with the procedures, and emergency physicians, professionals who need greater qualification.

## RESUMO

**Objetivo:**

Realizar um levantamento de dados na esfera penal envolvendo médicos no Tribunal de Justiça do Estado de São Paulo e estabelecer o número de processos penais que os envolveu, verificando o teor das acusações e a frequência das condenações, levando-se em consideração as especialidades em que eles atuavam.

**Métodos:**

Foi realizada pesquisa por palavra-chave na área de jurisprudência do *site* do Tribunal de Justiça do Estado de São Paulo com o termo “medical error” para as decisões proferidas de 1° de janeiro de 2011 até 31 de dezembro de 2016, sendo selecionado o assunto “direito penal”.

**Resultados:**

Foram encontrados e analisados 34 processos que corresponderam ao critério de inclusão. Houve 73,5% de condenação em primeira instância, observando-se discreta tendência de aumento no decorrer dos anos. O número de processos, de acordo com a especialidade médica, foi dez casos de emergência clínica, oito de obstetrícia, sete de cirurgia, três de pediatria (sendo que em um caso se referia a pediatria junto de obstetrícia), dois de ortopedia, dois de diretor clínico, um de anestesista e um de clínico não emergencista. Dentre estes casos, 6 se referiam à lesão corporal, 26 a homicídio e 2 a crime de desobediência.

**Conclusão:**

Os médicos mais expostos a erros médicos foram os das especialidades cirúrgicas, provavelmente devido ao maior índice de complicações associadas ao procedimento, e os emergencistas, necessitando de uma maior capacitação destes profissionais.

## INTRODUCTION

The functional purpose of criminal law is to protect the most important assets for the survival of society, so that penalties serve as a form of coercion, allowing the protection of the most relevant social assets, values and interests and, by applying the *ius puniendi*, *i.e* of the State to define crimes and enforce convictions,^(^ ^1^ ^)^ with criminal punishments regardless of the sanctions established by the civil law system.^(^ ^2^ ^)^

Among the indispensable rights, the most outstanding is the right to life, which is inviolable according to the Article 5 of the Brazilian Federal Constitution^(^ ^3^ ^)^ and ratified by Article 6, where it appears as a social right, together with health.^(^ ^3^ ^)^ Both, health and life are among the assets to be protected by Medicine, as provided by the Hippocratic Oath^(^ ^4^ ^)^ and by the Code of Medical Ethics from the Federal Council of Medicine (CFM – *Conselho Federal de Medicina*).^(^ ^5^ ^)^

Notwithstanding the foregoing, under the power of the media influence, in its capacity as a source of pressure about the use of health resources,^(^ ^6^ ^,^ ^7^ ^)^ patients are more and more filing the so-called medical lawsuits in the civil, criminal and administrative levels.^(^ ^8^ ^)^

Currently, the number of scientific studies on this subject is very inexpressive, with the prevalence of scientific products examining the scope of the administrative level of the regional professional councils.^(^ ^9^ ^–^ ^12^ ^)^ Therefore, the purpose of this work is to examine criminal medical lawsuits in the higher courts of the São Paulo State Court of Appeals.

## OBJECTIVE

To collect criminal justice data involving medical professionals in the São Paulo State Court of Appeals and to establish the number of criminal proceedings involving them, the content of the accusations and the conviction rate per specialty.

## METHODS

A keyword search was carried out in State Appellate Court case law^(^ ^13^ ^)^ website with the term “medical error” for decisions rendered from January 1^st^, 2011 to December 31^st^, 2016. The field “criminal law” was marked for subject. The following fields were also marked: appellate decision, higher court, *en-banc* court, ratification, and single-judge decisions.

The variables examined were definition of crime, defendants’ specialty, lower court conviction (yes or no) and year when the lawsuit was filed.

All data used were public and available on the internet. The project was submitted to the Ethics Committee and approved, with protocol 2.121.299, CAAE: 68109017.2.0000.5248.

## RESULTS

Seventy lawsuits were identified, 18 of which were eliminated because they were civil cases. Two were drug-trafficking cases unrelated to medical error, five were transit crimes, while one was an active corruption case involving a lawyer. Five proceedings were excluded because their defendants were not medical professionals. Two proceedings were excluded because they lacked data due to conflict of jurisdiction. Two proceedings were excluded due to repetition. Finally, 35 cases were available for analysis, but in fact only 34 were included in the sample, since we had no access to the data of one of them, probably because it was filed under seal.

Most cases resulted in the conviction of medical professionals by the lower court, totaling 73.5% convictions *versus* 26.5% acquittals by the lower court.

**Figure 1:**
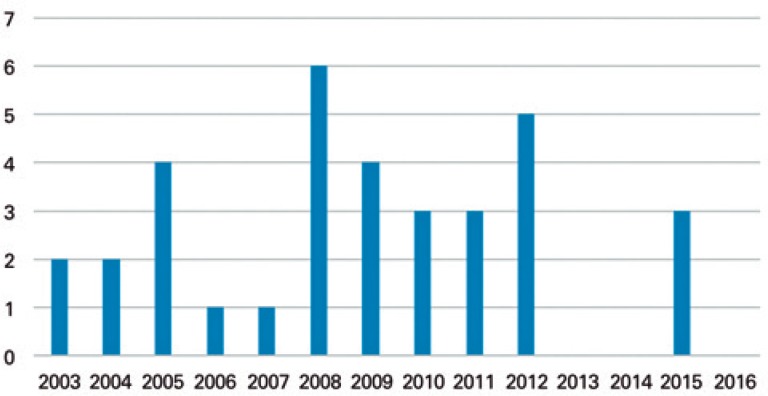
Proceedings per year of filing

A slight tendency towards increase was shown in this type of proceedings, according to the years they were filed (Figure 1).

Examined proceedings per medical specialty showed the following frequency: 29.4% were clinical emergency cases; 23.5% were gynecology-obstetrics; 20.5% were surgery (five cases of general surgery, one plastic surgery and one urologic surgery); 8.8% were related to pediatrics (one case involved also an obstetrician, which case was not listed in the previous item); 5.9%, orthopedic surgery; 5.9% involved clinical directors; 2.9% anesthesiologists; and 2.9% non-emergency internal medicine physician.

Accordingly, 55.9% of cases were related to surgical specialties, 35.2% clinical, 5.9% administrative specialty and 2.9% were related to clinical and surgical (gynecologyobstetrics and pediatrics) at the same time.

Considering only if the procedure or diagnosis was clinical or surgical, two cases about submission of patient's records were excluded, and of the 32 cases remaining, 71.9% were surgical and 28.1% clinical.

Among the definition of crimes, 5.9% were related to criminal contempt, 17.6% to bodily injury and 76.4% to homicide. No data were found on crimes typical of medical professionals, *i.e* perpetrated by physicians, such as violation of the duty of professional secrecy, failure to notify communicable disease, forged medical certificate, illegal exercise of Medicine, cases where medical professionals exceed the limits of their activities.^(^ ^14^ ^)^

Among clinical reasons or procedures that gave rise to claims, 2.9% of cases were related to administration of the wrong antivenin therapy; 2.9% to non-specified surgeries; 2.9% to liposuction; 2.9% to sling surgery for urinary incontinence; 5.9% to failure to submit patient's records to court; 17.6% to procedures related to child birth; 5.9% to hysterectomies; 2.9% to renal failure diagnosed as renal colic; 8.8% to trauma (mistreated traumatic brain injury; compartmental syndrome after fracture plastering; one scapula fracture with undiagnosed right hemothorax); 14.7% to abdominal surgeries (one cholecystectomy; one appendicitis surgery not performed; one undiagnosed appendicitis; one unspecified acute abdomen; one appendicectomy in which the anesthesiologist left the room before patient was fully recovered); 11.7% to infectious diseases (two undiagnosed cases of meningitis, in that, one was meningoencephalitis; one myocarditis, one leptospirosis identified as dengue fever); 2.94% to infarction and the Intense Care Unit assistance was not requested; 2.94% to complications of hip surgery due to iliac vein perforation; 2.94% to seizure in which the neurologist was not called; 2.94% to testicular torsion not diagnosed in emergency care; 2.94% to case of anaphylaxis after administration of benzathine penicillin; 2.94% to laryngectomy based on false histopathological report indicating malignancy; 2.94% to prostate resection with pudendal artery injury. Accordingly, the situations where the medical professional's fast urgent or emergent care was expected accounted to 94% of total cases.

## DISCUSSION

Firstly, the scope of this search was limited to the higher court case law, and due to the widely known delay of Brazilian courts to try claims, the scenario obtained was extremely extemporaneous. We also lost data due to website misclassification, and had to examine only 34 out of 70 cases.

Criminal cases represent a large occurrence in the courts. In a research conducted in the same website, choosing only criminal cases and removing the expression “medical error”, 155,395 decisions were found just for the year 2016.^(^ ^13^ ^)^

The increased number of proceedings, despite briefly commented in our work, is repeatedly shown in literature both at administrative^(^ ^8^ ^,^ ^10^ ^–^ ^12^ ^,^ ^15^ ^)^ and judicial levels.^(^ ^16^ ^)^

We were surprised at the high rate of conviction of medical professionals in this study. Owing to the slowness of the courts and the large number of possible appeals to stop the criminal action, we decided to evaluate only the lower-court data of each proceeding chosen for this variable. It is worth mentioning that as the case law database used in this study refers to the higher court information, and it is based on it that we have examined the lower court data, this is partially explained by the fact that defendants appeal when found guilty, creating a selection and detection bias in our data. Our analysis of the profile of civil actions in gynecology and obstetrics in the State of São Paulo showed a smaller number of decisions against the plaintiff than found for this scientific article.^(^ ^16^ ^)^ Studies involving the Court of Ethics of the Regional Medical Council (CRM - *Conselho Regional de Medicina*) of the States of Santa Catarina,^(^ ^17^ ^,^ ^18^ ^)^ Bahia,^(^ ^12^ ^)^ and Sergipe^(^ ^11^ ^)^ also showed higher rates of acquittal.

Sorting by specialties also followed the tendency of other studies, with surgery and gynecology-obstetrics accounting for the largest number of complaints^(^ ^8^ ^,^ ^11^ ^,^ ^16^ ^,^ ^19^ ^)^ Santos et al., in a study on ethical-professional proceedings in the State of Paraíba, showed higher prevalence of the same medical specialties as compared to the present study: gynecology-obstetrics, emergency, orthopedic surgery and surgery, anesthesiology, pediatrics and internal medicine.^(^ ^19^ ^)^ It is necessary to point out that due to situational and didactic reasons, internal medicine was included as emergency.

Delivery was deemed the tormentor of medical proceedings that went to court to seek justice. In fact, child birth has been shown as the main source of obstetrical complaints.^(^ ^16^ ^)^

Regarding the higher prevalence among surgical cases in comparison with clinical ones found, such data is compatible with the findings, despite the smaller statistical difference, in a descriptive study conducted in the three Chambers of the CRM of the State of Bahia, with administrative proceedings filed from 2000 to 2004.^(^ ^12^ ^)^

It is worth emphasizing that emergency situations, *i.e* expected from physicians, with the purpose of saving patient's life, corresponded to those described in most of the cases. The overload of urgency/emergency services is widely known, and that is partially due to the poor health care network.^(^ ^20^ ^)^ Despite resolution 1,451/1945^(^ ^21^ ^)^ and resolution CFM 2,077/2014^(^ ^22^ ^)^ having established the minimum number of medical professionals and their specialties for the provision of emergency care, in practice, there is no compliance with such number.

Although respiratory diseases have been shown as the main causes for emergency care,^(^ ^15^ ^)^ no action was filed involving this subject.

Among the types of criminal offenses, 5.9% of cases referred to criminal contempt; 17.6% to bodily injury and 76.4% to homicide. No data were found for abortion or failure to notify disease and forged medical certificate crimes. Silva et al., conducted a study in the CRM of State of Pará, where charges of homicide, bodily injury and damage to society's interest prevailed.^(^ ^15^ ^)^ On the other hand, Maia et al., found unintentional bodily injury as the crime with the largest number of proceedings filed with the Federal Prosecution Office Specialized in the Defense of Health (*Promotoria de Justiça Especializada na Defesa da Saúde*),^(^ ^23^ ^)^ in direct opposition to our data.

About criminal contempt in failing to submit patient's records, we emphasize the CFM's position, which provides in the book of medical ethics, as follows:^(^ ^5^ ^)^

Art. 73 of CFM: Physicians are prohibited from: disclosing any fact to which they had access by virtue of the exercise of their profession, unless for just reason, legal duty or consent, in writing by the patient. Sole paragraph. This prohibition prevails: a) even if the fact is of public domain or the patient has died; b) when physicians offer their deposition as a witness. In this case, the physician will appear before the authority and will inform his/her impediment; c) in the investigation of suspicion of crime the physician is prevented from disclosing a secret which may expose the patient to a criminal proceeding.^(^ ^5^ ^)^

## CONCLUSION

According to our studies, we could find out that the professionals more exposed to medical error were those working with emergency demands, probably due to the larger volume of medical care within a shorter period of time, particularly the surgical specialties, probably due to the higher index of procedure-associated complications.

The most probable explanation would be the existence of overload of urgency/emergency services, caused by a poor medical service infrastructure noncompliant with the resolutions issued by Councils, which determine a minimum number of physicians and their specialties to address emergencies.
